# Spatial Heterogeneity in Resource Distribution Promotes Facultative Sociality in Two Trans-Saharan Migratory Birds

**DOI:** 10.1371/journal.pone.0021016

**Published:** 2011-06-22

**Authors:** Ainara Cortés-Avizanda, Pablo Almaraz, Martina Carrete, José A. Sánchez-Zapata, Antonio Delgado, Fernando Hiraldo, José A. Donázar

**Affiliations:** 1 Department of Conservation Biology, Estación Biológica de Doñana, Consejo Superior de Investigaciones Científicas, Sevilla, Spain; 2 Department of Applied Biology, Universidad Miguel Hernández, Orihuela, Alicante, Spain; 3 Department of Physical, Chemical and Natural Systems, Universidad Pablo de Olavide, Sevilla, Spain; 4 Laboratorio de Biogeoquímica de Isótopos Estables, Estación Experimental del Zaidín, Consejo Superior de Investigaciones Científicas, Granada, Spain; University of Western Ontario, Canada

## Abstract

**Background:**

Migrant populations must cope not only with environmental changes in different biomes, but also with the continuous constraints imposed by human-induced changes through landscape transformation and resource patchiness. Theoretical studies suggest that changes in food distribution can promote changes in the social arrangement of individuals without apparent adaptive value. Empirical research on this subject has only been performed at reduced geographical scales and/or for single species. However, the relative contribution of food patchiness and predictability, both in space and time, to abundance and sociality can vary among species, depending on their degree of flexibility.

**Methodology/Principal Findings:**

By means of constrained zero-inflated Generalized Additive Models we analysed the spatial distribution of two trans-Saharan avian scavengers that breed (Europe) and winter (Africa) sympatrically, in relation to food availability. In the summering grounds, the probability of finding large numbers of both species increases close to predictable feeding sources, whereas in the wintering grounds, where food resources are widespread, we did not find such aggregation patterns, except for the black kite, which aggregated at desert locust outbreaks. The comparison of diets in both species through stable isotopes revealed that their diets overlapped during summering, but not during wintering.

**Conclusions/Significance:**

Our results suggest that bird sociality at feeding grounds is closely linked to the pattern of spatial distribution and predictability of trophic resources, which are ultimately induced by human activities. Migrant species can show adaptive foraging strategies to face changing distribution of food availability in both wintering and summering quarters. Understanding these effects is a key aspect for predicting the fitness costs and population consequences of habitat transformations on the viability of endangered migratory species.

## Introduction

The distribution of key resources affects the spatial structure and the social organization of animal populations [1]–[3]. Large aggregations of food may relax intraspecific competition, thus promoting the recruitment of individuals without any apparent adaptive value [3]–[5]. Nevertheless, most studies showing this link between animal distributions and resource availability are theoretical [4]. When empirical studies were performed, they were done at a reduced geographical scale (usually local) and/or from a monospecific approach [6], so generalizations beyond the population level (i.e., guilds or communities) are difficult to make.

Migratory species, from invertebrates to vertebrates, develop their life cycles in distant biomes, occupying wintering grounds far from their breeding areas [7]. Explanations proposed to understand migration include variation among species in dependence on temporally and spatially variable food resources [8]–[9], competitive ability [10]–[11] and life history traits [12]. Recently, some authors have suggested that behavioural flexibility, i.e. the ability of individuals to express distinct behaviours in different contexts through innovation and learning processes [13]–[14], might also influence the balance between migratory and resident strategies in environments with sharp seasonal changes [15]. Obligate migratory species are logically enforced to respond to variable conditions when moving from summering to wintering grounds. Within this scenario, however, the lack of studies tracking the response of migrant organisms to large-scale changes in the degree of heterogeneity in the spatial distribution of food resources is striking. Recent research suggests that animals, in particular birds, may develop specific behavioural strategies to compensate for the negative effects of environmental variability [16]–[17]. Thus, it seems important to discern whether migrant birds have flexible responses to variations in environmental conditions between summering and wintering grounds, such as changes in the availability of food resources. In addition, this information may be useful in understanding large-scale variability in the role of limiting factors on the viability of populations of migrant species of conservation concern [18]–[19].

Here, we examined the spatial response of individuals to changes in the distribution and availability of feeding resources between wintering and summering grounds using as a study system two long-lived migratory and facultative scavenger birds, the black kite *Milvus migrans* and the globally endangered Egyptian vulture *Neophron percnopterus*. These species have relatively similar foraging strategies and diets (relying on invertebrates and small to medium-sized vertebrates) and, although being territorial during breeding, they can feed together thus potentially competing for similar resources [20]–[21]. Previous studies of our monitored and other western European populations of Egyptian vultures and black kites indicate that individuals winter in the Sahelian region, between Senegal and Mali, as do the rest of the Western Palearctic populations [22], also overlapping their wintering areas. Throughout their annual cycles, these species exploit food resources that are strongly affected by human economies [23]. Specifically, in the European summering grounds, birds rely on rubbish dumps and supplementary feeding stations (so-called vulture restaurants) created after the prohibition of abandonment of carcasses derived from extensive livestock in the field [24]–[25]. In contrast, food availability in the African wintering areas is mostly unpredictable, since livestock is under an extensive regime and widespread in the field [23], with predictable carcasses only available at very few dispersed slaughterhouses in the vicinity of cities. In sub-Saharan biomes, moreover, there are particular emerging phenomena such as the outbreaks of desert locusts *Schistocerca gregaria*. This superabundant pulsed resource plays a key role in this arid ecosystem by providing food for some predators that aggregate in their surroundings [see 26 for details]. Under this framework, we specifically test whether the variability in the ecological scenarios that these trans-Saharan migrants encounter determine parallel or asymmetric responses in their distribution, abundance and trophic strategies in the two visited biomes. We hypothesize that the spatial distribution of our focal species will closely follow that of clumped resources in Europe, whereas in sub-Saharan Africa the species will be widespread, only showing aggregated distributions linked to pulsed resources (i.e. the desert locust outbreaks). According to the envisaged intraspecific differences in spatial aggregated distribution we predict that trophic overlap between the two species will be higher in their summering areas compared to wintering ones.

## Results

### Bird abundance and food resource availability

Egyptian vultures were detected in 20.6% of the African (n = 85) and in 35.2% of the European (n = 141) point counts, while the frequencies of detection of black kites were 34.7% and 50.7%, respectively. Large numbers of both Egyptian vultures and black kites feeding together were detected at predictable European feeding sources (i.e. rubbish dumps and vulture restaurants), reaching maximums around 73 and 143 birds, respectively. At those places, large numbers of another six different species were also found (see Table 1). At the few predictable African feeding sources (slaughterhouses), Egyptian vultures were absent and only black kites, reaching a maximum of 500 birds, and two raven species used them.

**Table 1:** Abundance of scavenger species (measured as the maximum number of individuals observed per day during all surveys) at European (n = 8) and African (n = 3) predictable feeding sources.

Species	Europe									Africa		
	Rubbish dumps					Vulture restaurants				Slaughterhouses		
***Neophron percnopterus***	**30**	**34**	**40**	**29**	**73**	**10**	**59**	**58**	**18**			
***Milvus migrans***	**55**	**41**	**0**	**143**	**100**	**26**	**6**	**1**	**4**	**500**	**300**	**45**
*Gyps fulvus*	100	20	0	0	35	505	259	357	522			
*Gypaetus barbatus*	2	0	0	0	0	0	0	0	0			
*Milvus milvus*	11	24	0	3	15	13	1	2	5			
*Aquila chrysaetos*	0	0	0	0	0	0	1	0	0			
*Buteo buteo*	1	0	0	0	0	0	0	0	0			
*Ciconia ciconia*	0	160	30	0	51	0	0	0	0			
*Larus cachinnans*	0	130	5	0	150	0	0	0	0			
*Corvus corax*	60	97	2	2	109	0	5	8	4			
*Corvus corone*	6	50	0	0	0	0	1	9	0			
*Corvus ruficollis*										55	1	1
*Corvus albus*										110	20	100
*Pica pica*	0	3	0	0	1	3	1	4	0			
**N survey days**	**6**	**8**	**4**	**2**	**8**	**7**	**12**	**8**	**7**	**3**	**2**	**2**

Focal species are shown in bold.

**Figure 1:**
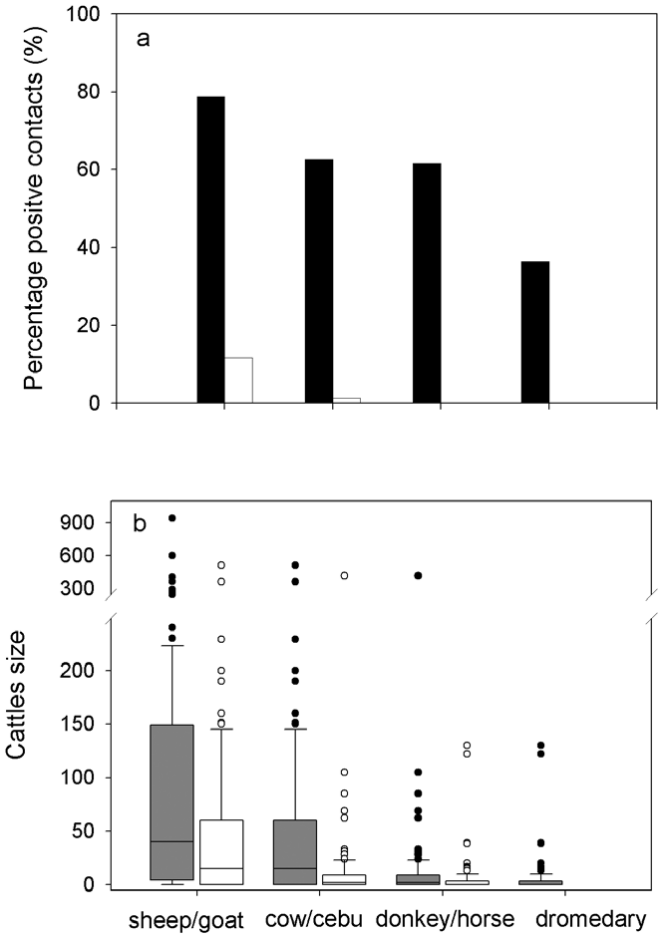
Cattle abundance a) (measured as percentage of positive contacts with cattle during road counts) and herd size b) in the summering (Europe; white) and wintering (Africa; grey) grounds of two trans-Saharan migrant species. Box plots of cattle sizes represent median values (horizontal line marks), the central 50% of the data (boxes), the range (whiskers) and outliers (dots).

Availability of food resources was different in summering and wintering grounds. In 91.9% (n = 99) of the African transects, we detected some kind of livestock while in Europe only 13% (n = 77) of transects contained livestock (χ^2^ = 107.144; *P*<0.0001) (Fig. 1). When we considered each livestock species separately, we also detected higher frequencies of presence in transects performed in wintering grounds (Africa: sheep and goat = 78.8%; cattle = 62.6%; horse = 61.6%; dromedary = 36.4%; Europe: sheep and goat = 11.7%; cattle = 1.3%; N×M exact Test; *P* = 0.0024).

### Food distribution, human presence and the spatial abundance of species

A preliminary analysis suggested no seasonal differences in the way that functional forms relate covariates to bird counts. Therefore, we pooled data in a single model for each species and spatial unit. The results of the fitting of each COZIGAM are shown in Table 2. The model selection approach consistently selected models including distance to the nearest feeding source and cattle for both species on the two continents.

**Table 2:** Relationship between species abundance and food distribution.

Species & season		Probability of Zero-inflation		Effect of covariates				
	% 0	*α* (± SE)	*δ* (± SE)	Spatial	Distance	Cattle	Slope	BIC^†^
**Egyptian Vulture**								
**Wintering**	78	1.251 (0.810)	−0.979 (0.717)	**21.320**	**−**	**−**	**−**	35.957
**Wintering**		5.642 (3.251)	**−4.173 (1.979)**	**−**	**6.302**	**7.718**	**−**	***5.189***
**Wintering**		0.600 (0.405)	0.034 (−)	**21.895**	**1.119**	**8.839**	**−**	81.327
**Summering**	65	4.195 (2.654)	−1.703 (1.194)	**28.910**	**−**	**−**	**−**	−6.841
**Summering**		−0.727 (0.653)	**1.639 (0.680)**	**−**	**2.97**	**−**	0.021 (0.010)*	**−** ***143.762***
**Summering**		**1.389 (0.349)**	3.271 (2.533)	**28.859**	**8.793**	**−**	−0.086 (0.044)*	58.645
**Black Kite**								
**Wintering**	64	0.301 (0.575)	−0.204 (0.186)	**26.310**	**−**	**−**	**−**	−280.850
**Wintering**		0.330 (0.458)	−0.273 (0.164)	**−**	**8.909**	0.006 (0.001) *	**−**	**−** ***446.654***
**Wintering**		0.459 (0.251)	−0.002 (−)	**28.063**	**8.256**	0.005 (0.001) *	**−**	−22.526
**Summering**	49	**1.052 (0.378)**	**−0.403 (0.197)**	**27.970**	**−**	**−**	**−**	−232.916
**Summering**		0.357 (0.378)	0.215 (0.231)	**−**	**4.752**	**−**	−0.059 (0.013) *	**−** ***321.818***
**Summering**		**1.408 (0.513)**	0.014 (0.243)	**27.605**	**8.183**	**−**	−0.119 (0.036) *	−97.737

Models were fitted using a COZIGAM with a Poisson link function and measuring the joint effects of distance to the nearest feeding source (distance) and human presence (slope in Europe and cattle in Africa) on the spatial abundance of Egyptian Vultures and black

kites in their summering (Europe) and wintering (Africa) grounds

Results for each spatial unit are shown for all seasons pooled in a single model. The table shows the proportion of 0 counts (% 0) in the dataset for each spatial unit. A linear model with a *logit* link function relates the probability of Zero-inflation (*p*_i_) to the covariates through the estimated spatial abundance in the GAM; in this model α is a constant and δ is a parameter measuring the homogeneity of Zero-inflation. The column for the “Effect of covariates” contains the estimated degrees of freedom (e.d.f) for each non-parametric term in the GAM, unless a parametric (functionally linear) term is selected; in these cases, denoted with the symbol ‘*’, the parametric estimate is shown instead. Statistically significant terms are shown in bold. Human density was measured as the density of cattle in Africa and the slope of the terrain in Europe. †The BIC denotes the Bayesian Information Criterion; the model minimizing this quantity is selected as the best descriptor of the dataset within the pool of fitted models, and is shown in bold type.

**Figure 2:**
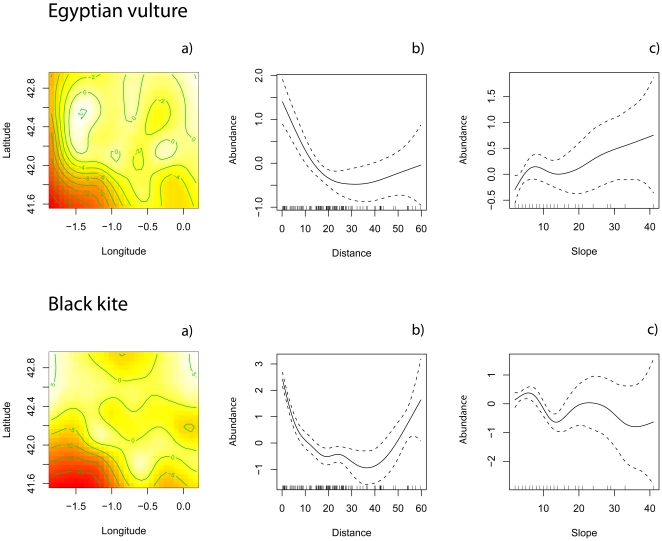
Plots of the additive terms (s (·) in equation 1) of the COZIGAM fitted to the Egyptian vulture (a–c) and black kite (d–f) in their summering grounds (Europe). In a) and d) the contour plot of the spatial effect in Eqn. 1, s(Latitude, Longitude), is shown. The coloured surface depicts the local probability density of the spatial distribution of counts, from low density areas (red) to high density areas (white). These colours correspond to areas with low and high bird density, respectively. The right diagrams show the partial residual plots for the effect of distance to the nearest feeding source (vulture restaurants and/or rubbish dumps; b, e) and human distribution (measured as the slope of the terrain; c, f) on the estimated spatial abundance of each species. The additive function is depicted as a solid black line, while the dotted lines show the 95% confidence intervals. For clarity, the location of each data point is presented as a rug plot along the bottom of each plot. Note that the terms have been scaled to have a 0 mean to **make the model identifiable** [**37**] **.**

**Figure 3:**
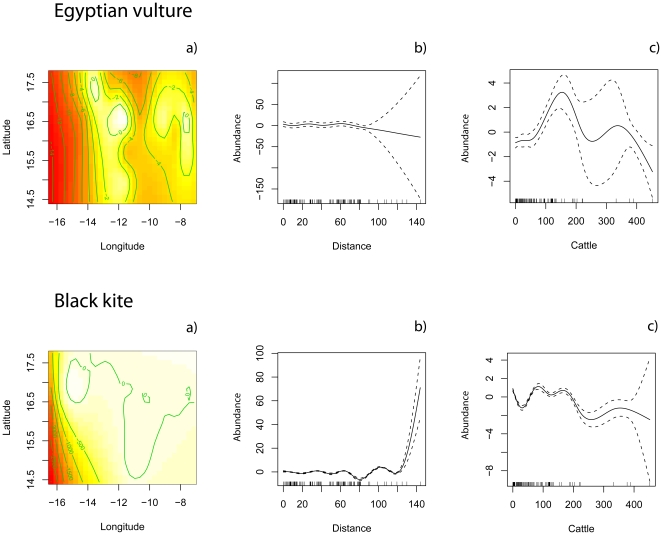
Plots of the additive terms of the COZIGAM fitted to the Egyptian vulture (a–c) and the black kite (d–f) in their wintering grounds (Africa). In a) and d) the contour plot of the spatial effect is shown. The right diagrams show the partial residual plots for the effect of distance to the nearest food source (nearest town with slaughterhouses; b, e) and the degree of humanization (cattle, measured as the number of livestock; c, f) on the estimated spatial abundance of each species. See Fig. 2 for further details.

Additionally, parametric (functionally linear) terms linked to human presence (*slope* in Europe and *cattle* in Africa) were selected for the black kite in both areas and for the Egyptian vulture in Europe. Although model structure was similar across species and continents, the profile of the non-parametric functions measuring the effects of the covariates on species abundance differed among continents, but not among species. Only for illustrative purposes, Fig. 2 and 3 depict the spatial non-parametric function fitted to the abundance of Egyptian vultures and black kites in Europe and Africa, respectively. Although these terms were not included in the final model (Table 2), they are useful for devising the spatial distribution of the focal species. After controlling for the effect of human presence in Europe (measured through the variable *slope*), the spatial abundance of Egyptian vultures and black kites decreases sharply with the distance to the nearest feeding source (Fig. 2). Note that this relationship is statistically significant (that is, the partial residual plots do not overlap 0) at distances lower than 40 km for both species. Thus, although it seems that the spatial abundance of both species increases at large distances to the feeding sources, the confidence limits widen as well (Fig. 2b and e). This effect is due to a single point in both figures. Moreover, the parameter measuring the homogeneity of Zero-inflation (*δ*) is positive for both species and statistically significant for the Egyptian vulture (Table 2), suggesting that the zero counts tend to accumulate at large distances from feeding sources.

**Figure 4:**
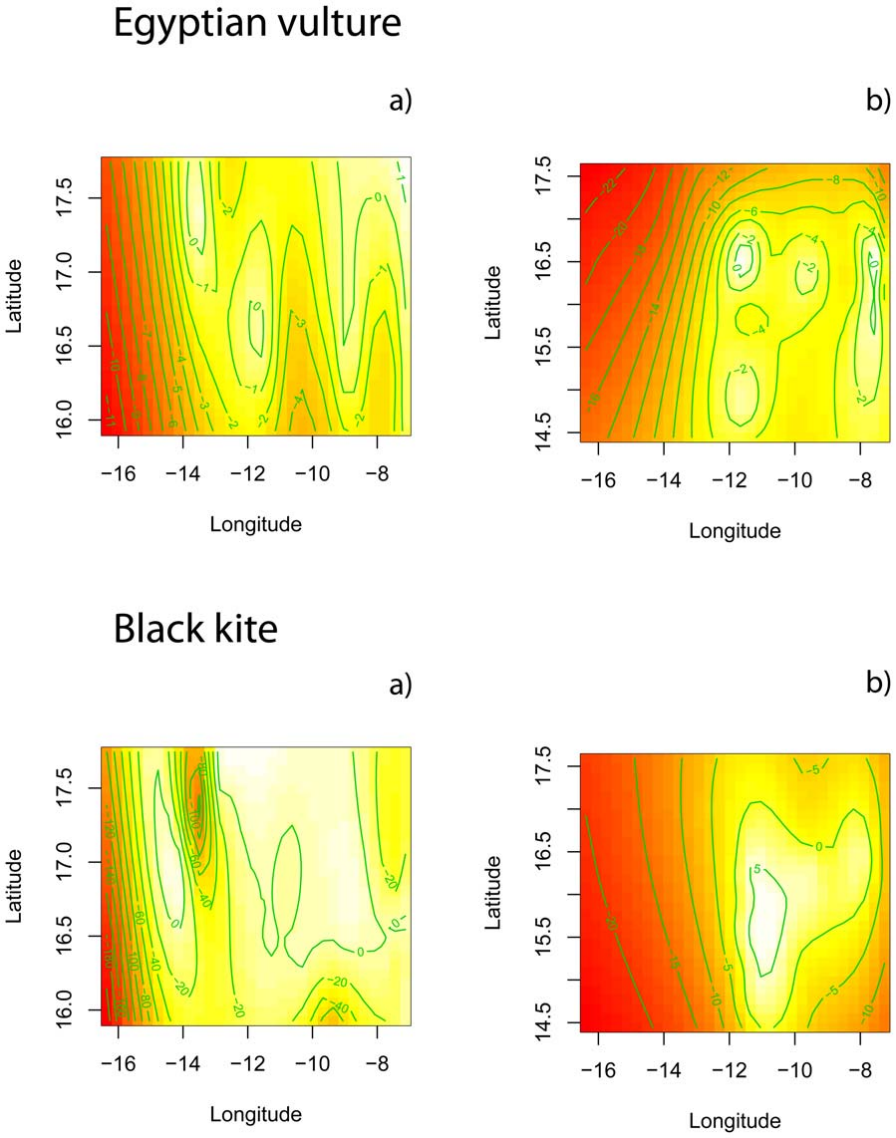
Contour plots of the spatial effect estimated by the COZIGAM fitted to the abundance of Egyptian vulture (a, b) and black kite (c, d) in Africa in two different years. For this plot only, the effect is shown for each species during the year without a locust outbreak (a, c) and the year with an outbreak (b, d).

In the wintering grounds, however, neither the distance to the nearest feeding source nor human presence (measured as abundance of cattle, *cattle*) seems to have an effect on the spatial distribution of vultures, although human presence was linked to the abundance of black kites (Fig. 3). Additionally, the proportion of zero counts was larger and the relative density of birds (n° birds/km^2^) was smaller than in Europe (Table 2), perhaps because the surveyed area was larger in Africa than on the other continent. It is thus worth noting that Fig. 3a depicts the location of only 18 birds in 11 groups scattered over a large area, so although the Egyptian vulture seems to be more aggregated in wintering grounds, we emphasize that the proportion of 0 counts was very high (78%) and the average group size was very small for this species (1.63 birds per contact). For the black kite, although the effect of the covariates was negligible for both years, the spatial distribution of birds was rather different in years with or without locust outbreaks. In Fig. 4, we plot the non-parametric terms for the spatial component of the abundance of each species in the years without (Fig. 4a, c) and with locusts (Fig. 4b, d). A shift in the spatial distribution of kites is evident, from the first (a scattered distribution) to the second year (an aggregation around the central portion of the study area, in which the population core of the locust outbreaks was located). Average group size increases accordingly, from 6.63 (±1.53) black kites per positive contact in the year without locusts to 32.47 (±10.84) birds in the year with the locust outbreak (*t*-test for samples with unequal variances, *t*_19_ = 2.09, *P* = 0.029). In the Egyptian vultures, there are no significant changes in bird abundances associated with desert locust outbreaks (1.63±0.34 in the year without locusts to 2.50±0.77 in the year with locusts; *t*_18_ = 2.10, P  = 0.317).

### Trophic overlap between species

**Figure 5:**
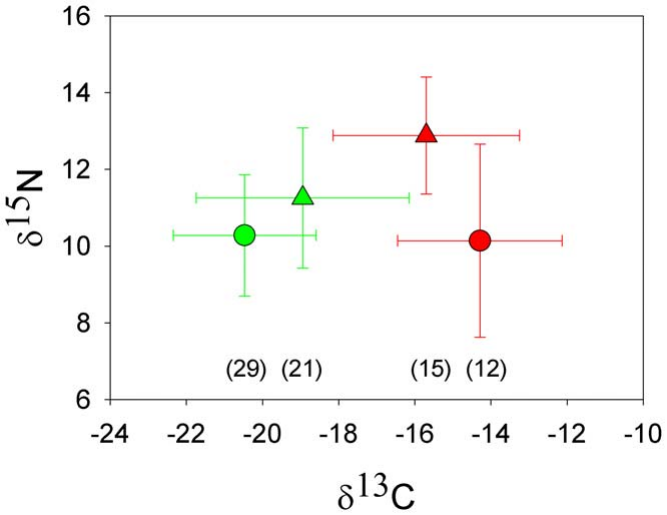
Stable carbon and nitrogen isotope values (mean ± SE, ‰) in feathers of Egyptian vultures (triangles) and black kites (circles) grown in their summering (Europe: green) and wintering (Africa: red) grounds. Numbers in brackets indicate sample sizes.

Isotopic signatures of feathers grown in Africa were different among species (Wilks' lambda  = 0.68, F_2,47_ = 10.81; *P*<0.001), but no interspecific differences were detected for feathers grown in Europe (Wilks' lambda  = 0.87, F_2,24_ = 1.73; *P* = 0.2; Fig. 5). Accordingly, when considering each isotope separately, significant interspecific differences were found among African samples, mainly regarding δ^15^N values (δ^13^C: F _1,48_ = 4.02; *P* = 0.051; δ^15^N: F _1,48_ = 18.8; *P*<0.001), while European samples did not show significant differences among species in the isotopic signatures (δ^13^C: F _1,26_ = 2.86; *P* = 0.1; δ^15^N: F _1,26_ = 2.2; *P* = 0.15).

## Discussion

### Food distribution, bird abundance and trophic overlap

Our results show how trans-Saharan migrant bird populations can exhibit different spatial arrangements depending on the food distributions found in their wintering and summering grounds. Sub-Saharan regions hold numerous and diverse herds of domestic livestock widespread in the field (see results for details). European areas, however, are long-term human-modified ecosystems where food resources for vertebrate scavenger species have been subject to different pressures and management decisions that have favoured their patchiness across years. As a consequence of these differences, the same birds must aggregate during summering, when food is clumped and patchily distributed, but can remain widely and near randomly distributed during wintering, when resources are widespread. But changes in bird distribution go beyond the species level, and interspecific trophic overlap also becomes apparent in the summering scenario of resource aggregation. Conversely, when both species track resources in the less managed habitats, they have the opportunity to find and exploit pulsed resources when possible. Indeed, during the desert locust outbreaks, the black kites shifted their spatial distribution in a conspicuous way, increasing group sizes markedly. In contrast, the spatial distribution of the Egyptian vulture did not change after the appearance of locust outbreaks.

These findings show that variability in the response to environmental changes differs between species, leading to scenarios with different degrees of resource partitioning, diet overlap and, consequently, interspecific competition and probability of coexistence [4], [28**]**–[****29]. Stable isotope analyses demonstrated that the diets of our focus species completely overlapped in European summering areas, whereas in the African wintering grounds, they were segregated. It is well-known that stable nitrogen isotope values show a stepwise enrichment with each trophic level [30]. In our case, there is a between-species difference in average δ^15^N (10.1 vs. 12.9) during wintering, indicating separated trophic levels. The analyses of pellets of the two species in the study area revealed that black kites relied on a mixed diet of arthropods and carcasses of domestic ungulates whereas Egyptian vultures consume more wild and domestic vertebrates (authors unpublished). Although the information is partial [31**]**–[****32] these diet differences probably also took place in Europe some decades ago within a scenario of healthier Mediterranean ecosystems, important extensive grazing and low number of predictable feeding points [33]. For its part, δ^13^C values show a slight enrichment with trophic level, but can reveal micro-habitat information on terrestrial ecosystems used due to the differential importance in the distribution of C3 and C4 plants [30], [34]. In our results, the marginally significant differences in stable carbon isotopes in African samples again seem to indicate a higher consumption by black kites of arthropods dependant on C4 herbaceous vegetation [26].

### On the adaptive value of changing grouping patterns

The above-described scenarios, where bird populations change their local abundances following resource distribution, raise a key question: are bird aggregations in European locations with predictable food adaptive or, on the contrary, a maladaptive result of the global scarcity of food and/or trophic distribution patterns? Clumped food seems to lead to high intra- and interspecific competition [25], [35**]**–[****36], which may negatively affect individual fitness [3], [6]. But on the other hand, the role of some aggregations (particularly roosts) in mate finding [37] and information transfer [38**]**–[****39] has been described. It should be taken into account, however, that although roosts and abundant food resources are frequently associated [33], selective forces leading to both kinds of aggregations may not be the same. In fact, at an interspecific level, individuals clump at feeding places but clearly separate for roosting even when the habitat requirements are very similar (pers. obs.), which lends evidence to a scenario of competition for food. At an intraspecific level, trade-offs between social costs and benefits could represent a challenge to properly managing endangered species. In our study model, for example, implications of changes in food distributions on aggregations would be different for vultures and kites, as the latter seem to be able to exploit different resources while the former, more endangered species, is less variable. Clearly, although the study of the adaptive value of living in groups is an old topic in ecology [40**]**–[****41] some main questions still remain unanswered.

In conclusion, our results show that the spatial distribution of food might not only affect the behaviour and success of local individuals [6], [41**]**–[****42], but can also shape the foraging strategies of entire populations, going beyond the species and potentially triggering consequences at multispecies levels. Future research should focus on the output of these strategies in the long-term population dynamics of migrant birds. In particular, it would be interesting to focus on the individual response to a changeable environment, as well as on its consequences at the population level. Populations composed of individuals behaving differently might have higher probabilities of success under changeable environments that those formed by more homogeneous individuals [43**]**–[****44]. Thus, under a scenario of global change, this approach can be useful in establishing probabilities of population persistence and conservation priorities.

### Conservation implications for trans-Saharan migrant birds

More than 50% of European birds are trans-Saharan migrants, and many of them show long-term population declines [19]. This is particularly true for some species like our focal Egyptian vulture, which is considered “globally endangered” [45]. Thus, the identification of potential limiting factors operating at wintering and breeding grounds is a key point to understanding their population trends [46**]**–[****47]. Recent studies suggest that mortality rates of migrant birds are mainly determined by factors operating at wintering grounds. In particular, survival rates of short and long-lived species, including the Egyptian vulture, have been positively associated with rainfall in the Sahelian region [48**]**–[****50]. Regarding our results, we found that foraging avian scavengers in Africa follow a distribution pattern very similar to that in which these scavenger species have co-evolved, i.e. environments where food resources are dispersed and sometimes appeared as pulsed events [51]. However, the Sahel region has degraded during recent decades as a consequence of a severe drought and human activities [23]. In this scenario, social and searching strategies -such as those described in this paper, could be scarcely efficient when food resources are highly depleted in space and/or time.

In the European breeding areas, landscape transformation and habitat loss have affected the availability of food resources, being considered of major concerns for the population viability of many predatory species [52**]**–[****53]. In fact, in Europe the few food resources available for avian scavengers are carcasses derived from intensive livestock, which are clumped and predictably disposed at supplementary feeding points [25]. Interspecific competition, which is enhanced in these situations, can promote the extinction of a species, even when it is a slow process not likely to be observed on the time scale of most scientific studies [54]. Besides ecological aspects of species and individual aggregations, other constraints such as the spread of illness, veterinary drugs and other contaminants [55**]**–[****56] are also negatively affecting species using these feeding points. Indeed, preliminary analysis shows that the detrimental effects of ingested antibiotics and the acquisition of pathogens at feeding points may decrease the health of vultures with a lethal potential, especially in nestlings and fledglings [57].

The relative importance of these negative factors operating during summer should be compared to that existing in the African wintering grounds in order to properly assess conservation criteria and priorities. Meanwhile, the appropriate management of trophic resources focused on reducing feeding costs for birds should be promoted. In this sense, future actions in Africa should focus on reducing the impoverishment of environments, avoiding landscape transformation by human overexploitation. In Europe, new management procedures should be implemented to generate a rather more heterogeneous pattern of food availability for birds, for example by promoting traditional, extensive agro-grazing practices [24], thus increasing individual health conditions and reducing intraguild competition.

## Materials and Methods

### Study areas

**Figure 6:**
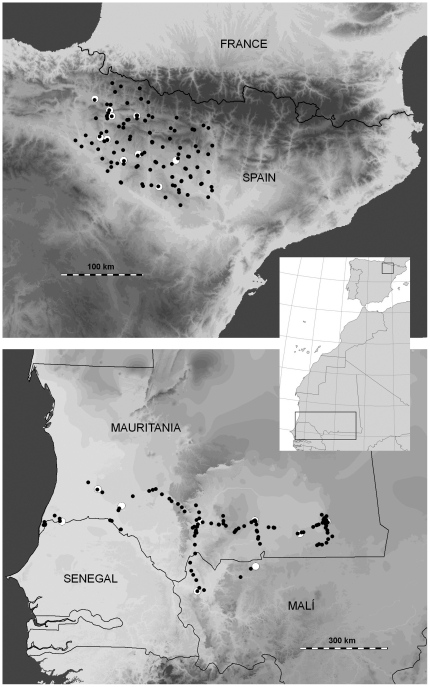
Maps of the studied areas with observation points (black dots) and predictable feeding sources (white dots).

Our study was performed in the Ebro Valley (Northern Spain) and in the Western Sahel region (Southern third of Mauritania and the adjacent areas of Senegal and Mali), covering the summering and wintering grounds of the Egyptian vulture and the black kite populations (Fig. 6). The first region extends over 10,000 km^2^ lying between the Pyrenees, the Iberian mountains and the Ebro Valley. The area has great orographic and climatic variation, with altitudes ranging between 300 to 2,400 m a.s.l. (Fig. 6). Human populations concentrate in valleys with large towns and villages [25]. This region holds one of the most important European populations of both avian scavengers [25], [48]. There are 380 breeding territories of Egyptian vultures (ca. 30% of the Iberian population) with several communal roosts where hundreds of birds regularly gather [25], [33]. Although no precise information is available for black kites, the species is abundant with more than 500 breeding pairs [58].

In the Western Sahel region, the relief is mostly barren with sparse rocky outcrops. Human population density is low (3 inhabitants/km^2^; United Nations World Population Prospects; http://esa.un.org/unpd/wpp/index.htm). Large concentrations of people inhabit a few cities such as Nouackchott, Kiffa, Aioun and Nema [26]. Nomadic shepherds inhabit temporary sparse settlements (Fig. 6). In this area there are no breeding populations of the two study species but large concentrations of Western Palaearctic migrants can be found [23]. Indeed, radiotracking studies carried out in Spain have shown that individuals summering in this area are wintering in the Sahel region, so we are confident that we are compiling information about factors affecting the same populations during summering and wintering. For further details on the study areas see [23], [26].

### Bird abundance and food resource availability

Following established methodologies by Sánchez-Zapata *et al.* [26], we surveyed the abundance of black kites and Egyptian vultures by means of 30-minute point counts (Africa: n = 42 in January 2004, and n = 43 in November-December 2004; Europe: n = 64 in May 2005, and n = 77 in July-August 2005; Fig. 6). Points were randomly distributed along both study areas, and were at least 10 km apart to avoid recounting birds. We recorded all the birds observed within a radius of 2 km. Additionally, for each point, we conducted 5-km car transects to determine the number of livestock: cattle *Bos primigenius*, sheep *Ovis aries*, goat *Capra hircus*, donkey *Equus asinus* and dromedary *Camelus dromedarius* in Africa and sheep, goat, cattle, horse *Equus ferus* and donkey in Europe. The presence and frequency of livestock species on the two continents were compared through Chi-square tests.

We also carried out censuses of scavenger birds at predictable feeding sources (three slaughterhouses in Africa, and four ‘vulture restaurants’ and five rubbish dumps in Europe) during 2004**–**2005. Visits lasted 30 min and on each occasion we recorded the maximum number of individuals observed.

### Food distribution, human presence and the spatial abundance of species

#### Field procedures

To determine whether predictable feeding sources influenced the spatial distribution of birds on both continents, we considered as a sample unit the abundance of Egyptian vultures and black kites at each observation point. As an explanatory variable, we considered the distance to the nearest predictable feeding source, i.e. nearest town with associated slaughterhouses in Africa, and vulture restaurants and/or rubbish dumps in Europe, calculated by the use of ArcView “Nearest feature” [59]. To control for the potential effects of humanization, we included the slope of the terrain (*slope*) at each count point performed in Europe (large towns are situated in flatter areas; [25]) and in Africa, the number of livestock (*cattle*) because in rural areas the size of the livestock population is linked directly with number of inhabitants [23]. We also took into account the availability of additional trophic resources in Africa such as locust outbreaks. During November-December 2004 there was an important outbreak covering broad regions of Mauritania, Mali, Senegal and Morocco. Before this event, the number of locusts was very low in the region [26]. Consequently, we distinguished counts performed during the desert locust outbreak (November**–**December 2004) from those obtained in previous seasons (January 2004) (see below for details of statistical procedures).

#### Spatial modelling of count data

Due to the nature of the survey, a large portion of sampling points contained a zero count on both continents, which yielded zero-inflated distributions with a larger proportion of zeros than expected from a standard Poisson count process [60]. Zero-inflated distributions are a *mixture* distribution in which a strong probability mass is located around 0 (the so-called 0-atom) and the remaining data behaves as a standard Poisson process, which gives rise to a regular distribution. During recent years several statistical approaches have been proposed for analyzing this particular distribution [60**]**–[****63]. Irrespective of the method, the standard procedure has been to analyze the mixture distribution in a two-stage approach. First, the response is dichotomized into zero and non-zero counts and analyzed using a presence/absence analysis. Then, a second analysis is conducted using only the non-zero data. It is well known, however, that the two-stage approach can very easily lead to conflicting results [64]. Thus, we used a recent and new method for ecological field data dealing with the excess 0′s while allowing us to test, in a robust manner, whether the differing spatial resource distribution among continents promotes different spatial patterns of bird distribution. The base model, proposed by Liu & Chan [64], uses a Generalized Additive Model (GAM), where no functional relationship is *a priori* assumed in the effects of the covariates on the response [65]. However, in our setting the response is assumed to follow a given distribution from the zero-inflated exponential family where the probability of zero-inflation is simultaneously supposed to be some monotone function of the expected response. Therefore, the mixture distribution is analyzed with a flexible, non-parametric model for the effects of some covariates on the response variable with the further, on-line constraint that the probability of zero-inflation in the response is a lineal function of the expectation. The resulting model is the so-called constrained zero-inflated Generalized Additive Model (COZIGAM) [64]. The constrained nature of the COZIGAM is useful when modelling spatio-temporal animal abundance data because the processes generating the 0 and non-0 inflation are linked through the same behavioural mechanisms [64].

To build the model, we consider that *y*_i_ is a count recorded in a random sampling point of the survey, and *x*_i_ is a value of the measured covariate. The mixture distribution, denoted by *h*(*y_i_*) is then defined as 
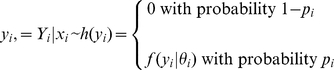



where the 0-atom models the zero inflation and the regular distribution (the non-zero portion) comes from an exponential family with probability density *f*(*y_i_* |*θ_i_*), in our case a Poisson distribution. A constraint is imposed so that the probability of zero-inflation, denoted by *p_i_*, is a monotone function of the expected values of the response variable, denoted by *μ_i_*. Using a logit link function, this restriction is written as 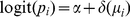
(1a)where *α* is a constant and *δ* is a parameter measuring the amount of homogeneity in zero inflation. Interestingly, if δ is found to be distinct from 0 this would indicate that the distribution of 0′s is not homogeneous, which means that a large proportion of zero counts accumulate along some gradient of the predictor variable. The response, with expected values denoted as *μ_i_* in Eqn. 1a, is modelled non-parametrically with a Generalized Additive Model [27]. For Europe, this model can be written in a simplified form as
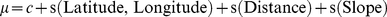
(1b)while for Africa it would be written as 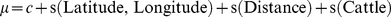
(1c)where *c* is a parametric constant and s (·) are the smooth functions measuring the non-parametric effects of covariates on the response. The term s(Latitude, Longitude) measures the non-parametric spatial component of the abundance survey [64]. Although a preliminary analysis with a non-parametric spline correlogram [66] suggested that the abundance data for both species lack spatial autocorrelation, we included the spatial term in eqn. 1c to check whether some spatial residual variation can be detected after estimating the covariate effects.

Eqns. 1b and c were fitted to the dataset for the corresponding spatial unit (Africa or Europe), and we further constructed alternative models by sequentially dropping the spatial term or the terms for the distance to the nearest feeding source and for human presence. For each covariate effect in eqn. 1 we tested whether a non-parametric (“wiggly”) function is preferred over a constant or parametric (“functionally linear”) one by testing if the estimated degrees-of-freedom (e.d.f.) of the non-parametric function deviates significantly from a pure parametric one, where e.d.f = 1 [27]. We measured the statistical performance of each model by subtracting the log-likelihood of each COZIGAM. We then calculated the Bayesian Information Criterion (BIC) of each fitted model [67]. This information criterion more heavily penalizes over-parameterized models with respect to alternative information criteria, such as the AIC. The model minimizing the BIC was selected as the best model. All the statistical analyses were conducted in R 2.11.1 [68], using the COZIGAM 2.0.3 package [64].

### Trophic overlap between species

#### Feather collection

We used stable isotope analyses to examine the diet overlap of our focal species in Africa and Europe. This methodology has been applied to studies on trophic relationships within vertebrate communities as well as to address specific questions regarding temporal and spatial variability in diets [69**]**–[****70]. Here, we performed stable isotope analyses of nitrogen (^15^N/^14^N, δ^15^N) and carbon (δ^13^C/^12^C, δ^13^C) from feathers [30], [71] collected on 41 black kite and 36 Egyptian vulture skins deposited in the Museum of Natural History of Madrid and in the Estación Biológica Doñana (CSIC). Focal species moult their feathers in wintering and in breeding regions [72**]**–[****73]. For this study, we only sampled skins of individuals collected in February-April, after their immediate arrival from wintering areas. From each skin, we sampled two feathers: one new, brilliant and with non-abraded edges, presumably grown in Africa, and another one older, with faded colour and with worn fringes presumably grown during the previous year in Europe. Very old feathers were not considered to avoid mixing samples of unclear origin.

#### Laboratory procedures

Stable carbon and nitrogen isotope assays were performed on 0.5 and 1 mg subsamples of feathers that were combusted in an elemental analyzer (Carlo Erba 1500NC) on-line with a Delta Plus XL mass spectrometer (EA-IRMS). Analysis of δ^13^C and δ^15^N were in triplicate. The overall precision of analyses was ±0.1 ‰ for δ^13^C and δ^15^N. The stable isotope composition is reported as δ values per mil: δ =  (R_sample_/R_standar_ –1) *1000, where R = ^13^C/^12^C for δ^13^C or ^15^N/^14^N for δ^15^N values. The international reference standard for ^13^C/^12^C is PDB (Pee Dee Belemnites, a fossil marine carbonate of biogenic origin) and for ^15^N/^12^N in the AIR (average of Atmospheric Air).

#### Analytical procedures

For both carbon and nitrogen, differences in isotopic signatures between the two study species within each biome were tested first for the two isotopes combined (MANOVA), then for carbon and nitrogen taken separately (one-way ANOVA) [74]. Values are expressed as means ± SE. The statistical software used was SPSS version 17.0.
